# Highly Efficient Nanoscale Analysis of Plant Stomata and Cell Surface Using Polyaddition Silicone Rubber

**DOI:** 10.3389/fpls.2019.01569

**Published:** 2019-12-13

**Authors:** Yi He, Kaiyue Zhou, Zhemin Wu, Boxiu Li, Junliang Fu, Chinho Lin, Dean Jiang

**Affiliations:** ^1^State Key Laboratory of Plant Physiology and Biochemistry, College of Life Sciences, Zhejiang University, Hangzhou, China; ^2^State Key Laboratory of Subtropical Silviculture, Zhejiang A&F University, Hangzhou, China; ^3^Sir Run Run Shaw Hospital, College of Medicine, Zhejiang University, Hangzhou, China; ^4^Second Affiliated Hospital of Zhejiang University, Zhejiang University, Hangzhou, China; ^5^Department of Life Sciences, National Chung Hsing University, Taichung, Taiwan

**Keywords:** stomata, stomatal aperture, cell surface, plant surface impression technique, nano-scale

## Abstract

Stomata control gas exchange and water transpiration and are one of the most important physiological apparatuses in higher plants. The regulation of stomatal aperture is closely coordinated with photosynthesis, nutrient uptake, plant growth, development, and so on. With advances in scanning electron microscopy (SEM), high-resolution images of plant stomata and cell surfaces can be obtained from detached plant tissues. However, this method does not allow for rapid analysis of the dynamic variation of plant stomata and cell surfaces *in situ* under nondestructive conditions. In this study, we demonstrated a novel plant surface impression technique (PSIT, Silagum-Light as correction impression material based on A-silicones for all two-phase impression techniques) that allows for precise analysis of plant stomata aperture and cell surfaces. Using this method, we successfully monitored the dynamic variation of stomata and observed the nanoscale microstructure of soybean leaf trichomes and dragonfly wings. Additionally, compared with the analytical precision and the time used for preparing the observation samples between PSIT and traditional SEM, the results suggested that the analytical precision of PSIT was the same to traditional SEM, but the PSIT was more easy to operate. Thus, our results indicated that PSIT can be widely applied to the plant science field.

## Introduction

Stomata are microscopic pores found in large numbers on the epidermal surface of most aerial parts of higher plants, and they have been documented in fossil records as early as the late Silurian era, nearly 411 million years ago (Lawson et al., 2009; Edwards et al., 1998). Stomata are central to the physiology of land plants, as environmentally induced changes in stomatal development and movements have profound effects on gas exchange between the atmosphere and the leaf (Jiang et al., 2012; Webb and Baker, 2002). Stomata are formed by pairs of guard cells and function as gateways for controlling gas exchange and transpirational water loss. Stomatal opening promotes plant growth by enhancing carbon dioxide uptake and transpirational water loss, which are both essential for photosynthesis and nutrient uptake from the soil to the plant body, respectively (Gray et al., 2000; Murata et al., 2015; Jezek and Blatt, 2017). The number and distribution of stomata also affect gas exchange and are closely regulated and coordinated with cell growth and division, while they also preserve a level of plasticity to respond to ever-changing environmental conditions (Pilliteri et al., 2012). The important biological functions of stomata thus render them a focus of great interest for plant biologists.

At present, although SEM has been used for high-resolution imaging of plant cell surfaces (Faulkner et al., 2008; Talbot and White, 2013), specimen preparation for traditional SEM is complicated and time consuming. In previous stomata studies, several types of materials have been used to create leaf impressions. In 1961, Sampson described a two-stage process using an early silicone rubber and clear nail polish, which yielded a positive impression of biological surfaces suitable for microscopical observation. Gloser (1967) experimented with a solution of Perspex (methyl-methacrylate) in chloroform, and Jones (1992) and Kagan et al. (1992) used nail polish to study stomata (Weyer and Johansen, 1985; Poole et al., 1996; Geisler et al., 2002) and the growth of the leaf epidermis (Elsner et al., 2012), respectively. These techniques were later evaluated for measurements of stomatal aperture. Furthermore, William and Green, (1988) and Geisler et al. (2000) analyzed plant meristem growth and the behavior of cells over time using sequential dental resin impressions. In addition, as a classical technique in stomatal research, epidermal peels have allowed for the analysis of stomatal responses in the absence of mesophyll and have contributed to important breakthroughs in stomatal research (Zeiger, 1983). However, these techniques also share the common disadvantage of involving organic solvents, material viscosity, environmental humidity, and mechanical damage to affect the results (Gloser, 1967; Lin et al., 1977; Zeiger, 1983; Weyer and Johansen, 1985; William et al., 1987; William and Green 1988; Kagan et al., 1992; Poole et al., 1996; Zhou et al., 2016). Furthermore, the above methods could not provide high-resolution and high-throughput images that would permit the analysis of stomatal aperture and density in an intact plant specimen. Therefore, this study aimed to develop a new technique called the plant surface impression technique (PSIT) to study the plant stomata and cell surface. Compared to conventional methods, our method confirmed nanoscale structures of plant and animal cell surfaces at about 50 nm, which is the precision limit of the impression technique.

## Material and Methods

### Plant Growth Conditions

The salt-sensitive cultivar Melrose (*Glycine max*) and the salt-tolerant line S111-9, a stable line selected from somatic hybrid descendants of wild salt-tolerant ACC547 (*G. cyrtoloba*; Yang et al., 2007), were used in this study. When the first pair of leaves was fully expanded, the seedlings were transplanted into complete nutrient solution and cultured at 25/22°C (12 h light/12 h dark) with approximately 70% relative humidity in a growth chamber. For experiments, 25-day-old plants (fourth leaf stage) were selected and transferred to a complete culture solution (He et al., 2014; Wang et al., 2015) with 0 or 150 mM NaCl. The third fully expanded leaves were used for all analyses.

Tobacco (*Nicotiana tabacum*) and Arabidopsis (*Arabidopsis thaliana* ‘Col-0’) plants were grown in soil at a 12 h light (28°C)/12 h dark (25°C) cycle and 12 h light (22°C)/12 h dark (20°C) cycle with 60% humidity in growth chambers, respectively. Wild type (WT) rice plants (*Oryza sativa* ‘Nipponbare’) were grown in hydroponic solution using standard methods as described (Kang et al., 2013) under a 12 h light (30°C)/12 h dark (26°C) photoperiod with 70% humidity. Further, 25-day-old plants were used for stomata analyses using the plant surface impression technique (PSIT).

### Gas Exchange Measurement

Gas exchange was measured with a portable photosynthesis system (*Licor-6400*; LICOR Inc., Lincoln, NE, USA) equipped with a red blue LED light source. All measurements were carried out at a photon flux density (PFD) of 1200 µmol m^-2^ s^-1^, a leaf temperature of 25°C, and CO_2_ of 400 ± 5 µmol mol^-1^ in the sample chamber (He et al., 2014). Each experiment was repeated three times in different plants.

### Preparation of Leaf Mold and Cast

A soybean-sized (the diameter is 1 cm) portion of the base and catalyst (Silagum-Light; DMG company, Germany, Cat: REF 909713) was mixed at a 1:1 ratio in a left-to-right motion about 10 times, avoiding air bubbles. The mixture was placed on the leaf surface and allowed to harden for approximately 5 min, creating the negative mold. Subsequently, the mold was removed after hardening and examined to ensure there were no air bubbles, contaminants, or cracks on the leaf surface.

To prepare the cast (also called the positive mold), an adhesive and hardener (NAN PAO epoxy 906; NANPAO resins chemical company; Cat: 906) was used at a 1:1 ratio, and this was mixed approximately 10 times. A droplet of the epoxy resin was placed in the hardened molds. Next, the cast and mold were placed in the baking box (60°C) for 1 h to allow the cast to harden completely. Finally, the hardened cast was used for SEM observation.

### SEM Observation

The casts were coated with gold for 120 s at 20 mA in a E-1010 ion sputter (Hitachi), then observed and photographed in a variable-pressure SEM (HITACHI UHR FE-SEM SU8010; Hitachi Ltd, Japan) with a beam accelerating voltage of 10 kV and 400×–100,000× magnification.

### Statistical Methods

All assays described above were repeated at least three times on three biological replicates. For multiple comparisons, the data were first examined by one-way analysis of variance (ANOVA) to assess the equality of variance (*Levene-test*), followed by Tukey’s multiple comparison tests to determine significant differences (*P* < *0.05*) of the mean, using the SAS 8.0 statistical software package (SAS Institute, Inc., Cary, NC, USA) (He et al., 2014).

## Results

### Characterization of Silagum-Light and Adhesive

**Figure 1:**
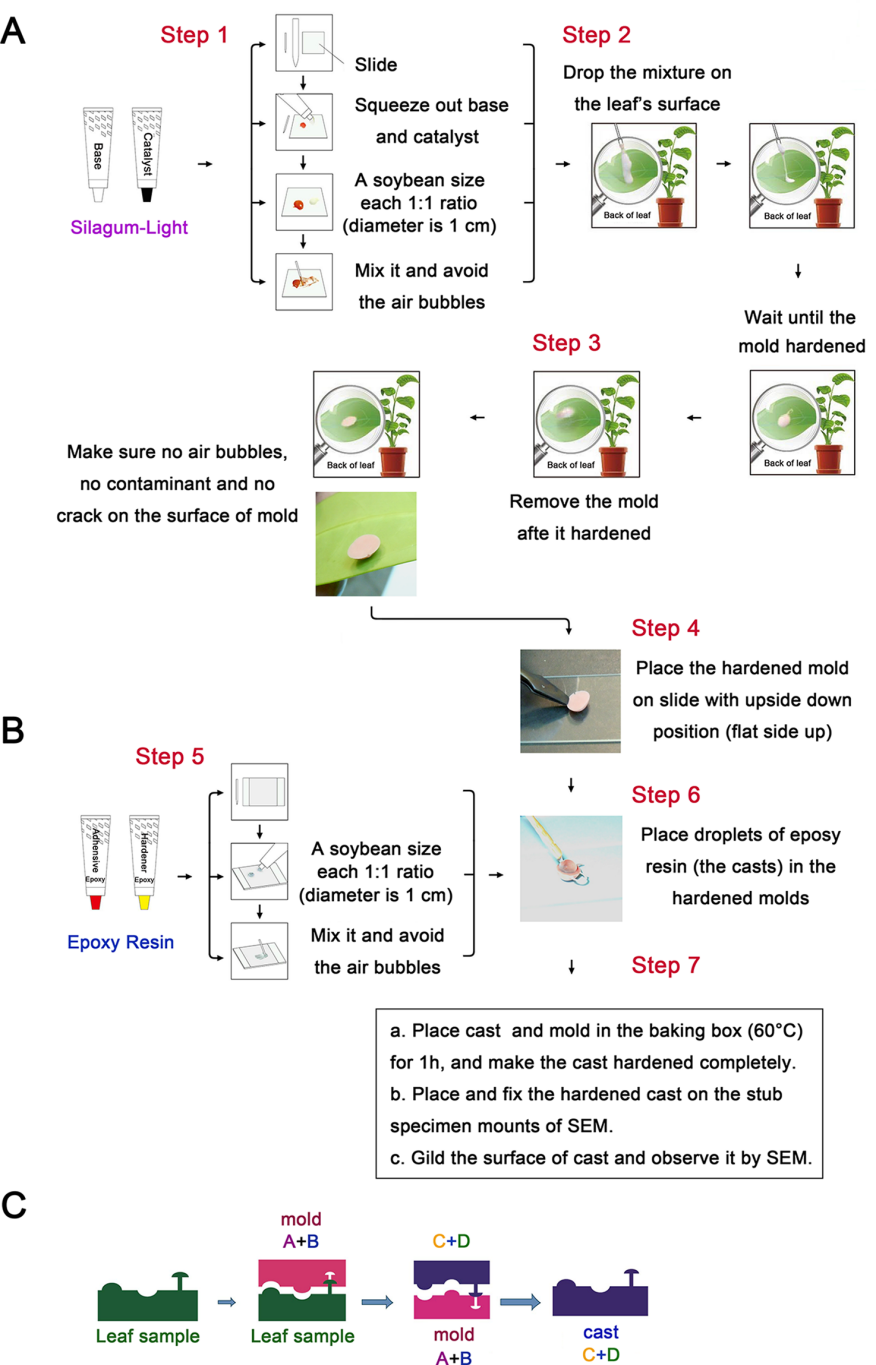
Schematic illustration of the plant surface impression technique (PSIT). **(A)** The procedure for mold preparation using Silagum-Light. The base and catalyst were added at a soybean size (the diameter is 1 cm) (1:1 ratio) on the slide and mixed in a left to right motion about 10 times, avoiding air bubbles. Subsequently, the mixture was placed on the leaf’s surface and allowed to harden for approximately 5 min. After hardening, the mold was removed and placed on the slide in an inverted position. **(B)** The procedure for cast preparation using NAN PAO epoxy 906. A soybean size (the diameter is 1 cm) of adhesive and hardener (epoxy resin) was mixed about 10 times, avoiding air bubbles. Following this, droplets of the epoxy resin were placed in the hardened molds. The cast and mold were placed in a baking box (60°C) for 1 h to allow the cast to harden completely. Finally, we placed and fixed the hardened cast on the stub specimen mounts for SEM and gilded the cast surface for SEM observation. **(C)** The principle of plant surface impression technique. This picture illustrates the principle of mold and cast preparation using Silagum-Light (polyaddition silicone rubber) and epoxy resin (NAN PAO epoxy 906), respectively.

To obtain suitable materials to prepare the negative and positive molds, the Silagum-Light and NAN PAO epoxy 906 adhesive were used. The Silagum-Light (DMG company, Cat: REF 909713), as correction impression material, is based on A-silicones for all two-phase impression techniques. The main ingredients of Silagum-Light are addition-curing vinyl polysiloxanes, hydrogen polysiloxanes, fillers, pigments, additives, and platinum catalyst. Silagum-Light (Type A polysilicone rubber) is formed by addition polymerization, which is a double bond reaction. This reaction does not produce a by-product, further ensuring precision of the impression model size. Furthermore, Silagum-Light exhibits non toxicity, low viscosity, good hydrophilicity and fluidity, excellent morphological stability, sufficient hardness, precise details, and surface recovery capacity characteristics. In addition, Silagum-Light is easy to operate for the preparation of molds (**Figure 1**), which can be stored for extended periods; the precision of the mold would not be affected even when stored at room temperature for several months. These characteristics render Silagum-Light suitable for creating the negative mold of the plant stomata and cell surfaces.

In our experiment, NAN PAO epoxy 906 adhesive (NANPAO resins chemical company, Cat: 906) was used to create the positive mold. This adhesive is a two-component epoxy resin (named epoxy resin AB glue), which is a high temperature tolerant glue that can harden in a short time. The epoxy resin AB glue also exhibits good fluidity, excellent morphological stability, precision for capturing detail, and surface recovery capacity. Furthermore, when hardened, it can easily separate from the negative mold, which is important to ensure the precision of the positive mold. Moreover, the positive mold can be stored for a very long time (2 years at least).

### Plant Surface Impression Technique Procedure

To further clarify the process of the plant surface impression technique (PSIT), a schematic of the PSIT procedure is depicted in **Figure 1**. Silagum-Light was used to create the negative mold (**Figure 1A**). First, a soybean-sized (the diameter is 1 cm) (1:1 ratio) amount of the base and catalyst was placed on the slide and was mixed using a left-to-right motion about 10 times, avoiding air bubbles. Second, the mixture was dropped on the surface of the plant leaf and allowed to harden for about 5 min. Finally, after hardening, the mold was removed and placed on the slide in an inverted position, completing negative mold formation. For the positive mold, NAN PAO epoxy 906 was used to create the cast (positive mold) (**Figure 1B**). First, a soybean-sized (the diameter is 1 cm) portion of the adhesive and hardener (epoxy resin) was mixed quickly about 10 times, ensuring no bubbles formed in the mixture. Next, droplets of the epoxy resin were placed in the hardened molds (**Figure 1C**), and then the cast and mold were placed in the baking box (60°C) for about 1 h until the cast hardened completely. Finally, the hardened cast was placed and fixed on the stub specimen mounts of a Scanning Electron Microscope (SEM), and the cast surface was gilded for observation. A patent for the PSIT method was acquired in 2015 (ZL201510071346.5; China).

### Plant Surface Impression Technique to Study Stomata of Soybeans

**Figure 2:**
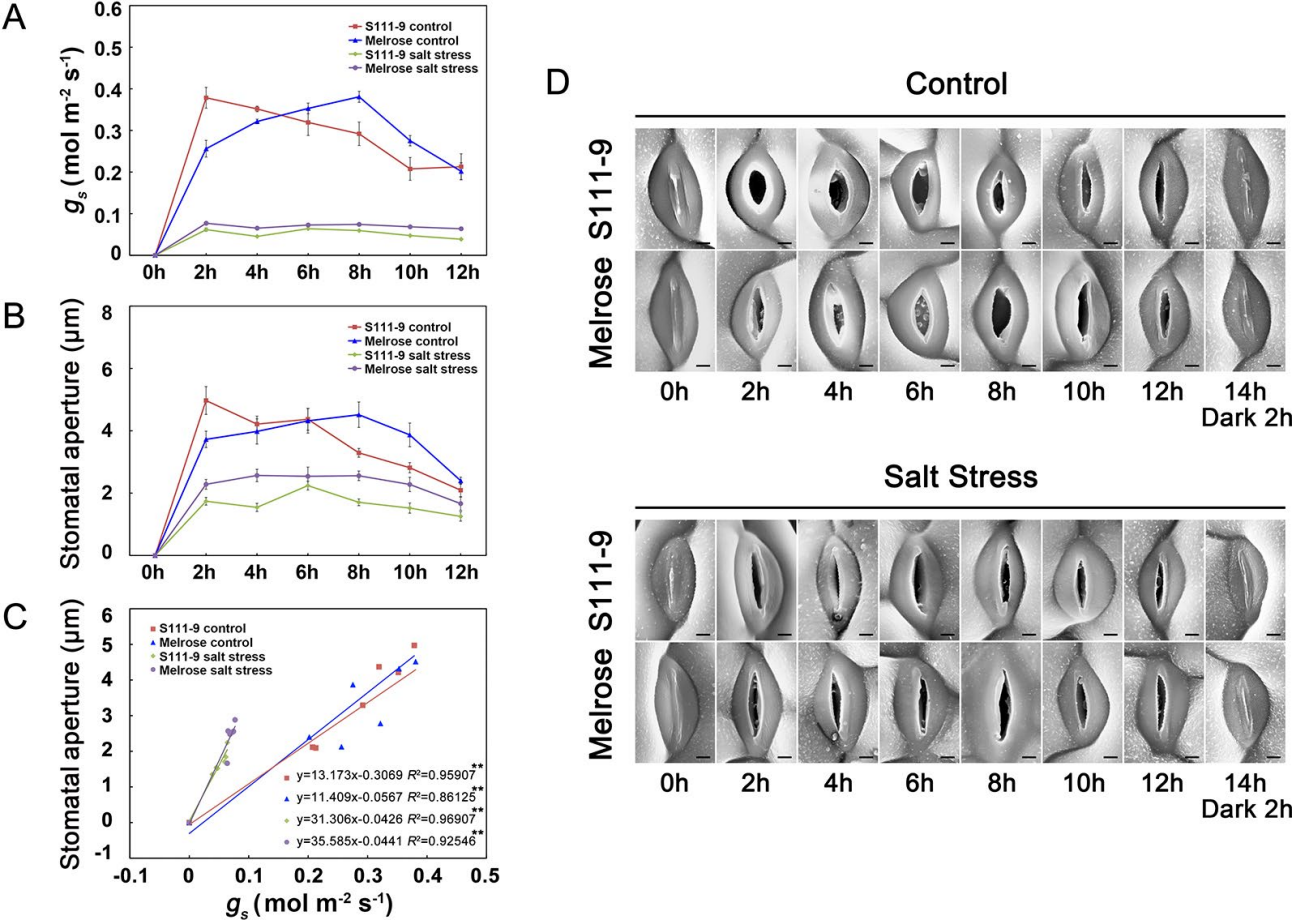
Application of the plant surface impression technique (PSIT) to study stomatal aperture and the correlation between stomatal conductance and stomatal aperture. The third fully expanded leaves of 25-day-old Melrose and S111-9 under no salt stress and 150 mM salt stress for 0, 2, 4, 6, 8, 10, and 12 h were used in this experiment. **(A)** Effect of salt stress on stomatal conductance (g_s_). The g_s_ were measured with a portable photosynthesis system (*Licor*-6400; LICOR Inc., Lincoln, NE, USA) equipped with a LED red blue light source. All measurements were carried out at photon flux density (PFD) of 1,200 μmol m ^-2^ s^-1^, a leaf temperature of 25°C, CO_2_ of 400 ± 5 μmol mol^-1^, and relative humidity of 70% in the sample chamber (mean ± SD, n = 3). **(B)** Effect of salt stress on stomatal aperture. SEM images at 1,500× magnification by the PSIT were used for stomatal aperture determination, which were measured by Image J software. In total, 40 stomata were measured from at least three leaves of independent seedlings at each time point, and these were repeated at three biological replicates for statistical analysis (mean ± SD, n = 3). **(C)** Correlation analysis between g_s_ and stomatal aperture under the control or salt stress condition. The analysis data were obtained from **(A**, **B)**. **(D)** Effects of salt stress on stomatal aperture in both soybean varieties (S111-9 and Melrose) by the PSIT. All sections were observed under SEM at 1,500× magnification. Scale bar = 3 μm; n refers to number of biological replicates. ** - extremely significant difference (*P* < 0.01).

To investigate the precision of the plant surface impression technique (PSIT), we used a portable photosynthesis system (*Licor*-*6400*) to measure the stomatal conductance (*g_s_*) of two varieties of soybean (Melrose and S111-9) under no salt stress and 150 mM salt stress conditions when the plants were exposed to light (He et al., 2014) (**Figure 2**). Furthermore, we used the PSIT to obtain several images of stomata under the same conditions, using Image J software to examine stomatal aperture (**Figures 2B, D**). The results showed that the variation tendency of *g_s_* was in accordance with the change in stomatal opening, as measured by the PSIT (**Figures 2A, B**) under no salt stress or 150 mM salt stress conditions. Although there was a significant decrease in *g_s_* under 150 mM salt stress, their variation tendency was still in accordance with stomatal aperture. Moreover, the regression analysis showed that there was a significant positive correlation between *g_s_* and stomatal aperture under no salt stress and 150 mM salt stress conditions (**Figure 2C**). In addition, the stomatal throat (the narrowest part of the stomatal pore often lies well below the cuticular ledges of a stomatal complex) could be clearly observed using the PSIT (**Figure 2D**). Although Weyers proposed the notion of the stomatal throat in 1985 (Weyers and Johansen, 1985), few techniques have allowed the observation of this stomatal component. Further, because of the extreme low viscosity of Silagum-Light, the PSIT did not affect or damage the plant surface and stomata. Therefore, the PSIT is highly suitable for monitoring real-time changes in the stomatal aperture and can monitor stomatal aperture *in situ* changes over time. The results *in situ* even more accurately reflect daily variations in stomatal openings.

**Figure 3:**
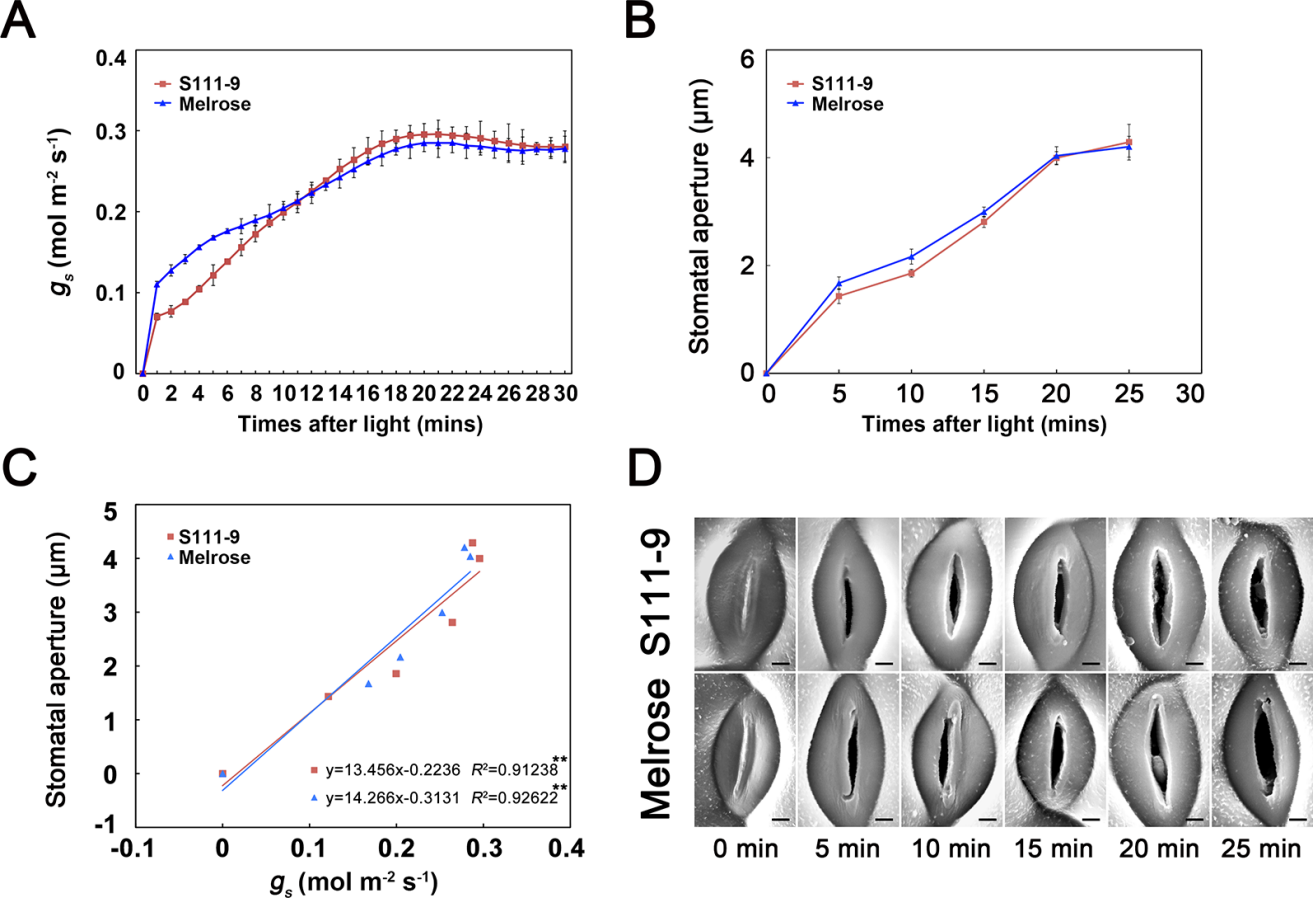
Application of the plant surface impression technique (PSIT) to study the dynamic variation in stomatal aperture and the correlation between stomatal conductance and stomatal aperture after light illumination. The third fully expanded leaves of 25-day-old Melrose and S111-9 under no salt stress for 0, 5, 10, 15, 20, and 25 min were used in this experiment. **(A)** Changes in stomatal conductance (g_s_) after light illumination. The measurement method for g_s_ was the same as described in **Figure 2**. **(B)** Changes in stomatal aperture after light illumination. The determination of stomatal aperture was described in **Figure 2**. **(C)** Correlation analysis between g_s_ and stomatal aperture. The analysis data were obtained from **(A**, **B)**. **(D)** Changes in stomatal aperture in both soybean varieties (S111-9 and Melrose) by plant surface impression technique. All sections were observed under SEM at 1,500× magnification. Scale bar = 3 μm. Three biological replicates were completed in this experiment. ** - extremely significant difference (*P* < 0.01).

To further test whether the PSIT can be used to monitor changes in stomatal aperture status from closed to open when the leaf is exposed to light, we measured the dynamic variation of *g_s_* and stomatal aperture at 30 min (**Figure 3**). The results indicated that *g_s_* quickly increased and reached about 0.3 mol m^-2^ s^-1^ in the first 30 min after the leaf was exposed to light (**Figure 3A**). Additionally, the stomatal aperture of Melrose and S111-9 quickly increased from 0 to about 4 µm (**Figures 3B, D**), in accordance with the variation of *g_s_*. The regression analysis indicated a significant positive correlation between *g_s_* and stomatal aperture in two soybean varieties (**Figure 3C**). These results suggest that the PSIT can be used to analyze daily variations in stomatal aperture status and to precisely analyze the short-time dynamic variation of stomatal aperture *in situ* for 30 min.

### Application of the PSIT to Study the Cell Surface

**Figure 4:**
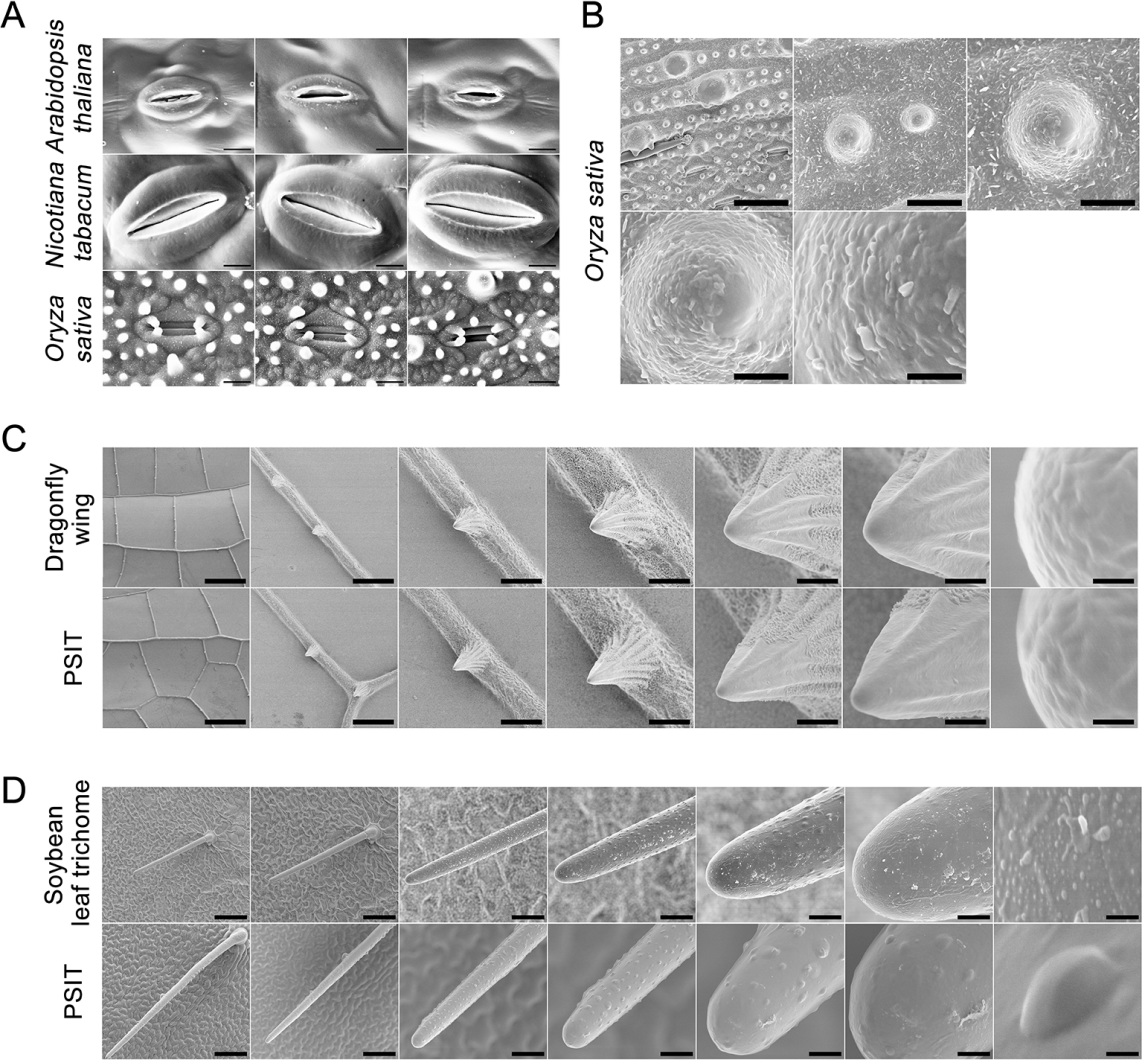
Application of plant surface impression technique of the (PSIT) in Arabidopsis, tobacco, rice, soybean, and dragonfly. **(A)** The stomatal aperture of Arabidopsis, tobacco, and rice were determined by the PSIT. All sections were observed under SEM at 2,000× magnification. Scale bar = 20 μm. **(B)** Protrusions of the rice leaf were determined by the PSIT. The sections were observed under SEM at 1,000×, 5,000×, 10,000×, 20,000×, and 50,000× magnification, with 25, 5, 2.5, 1.25, and 0.5 μm scale bars indicated, respectively. **(C)** The structure of the dragonfly wing. The surface structure of the dragonfly wing was observed under SEM using dragonfly wing tissue and PSIT. From left to right, the magnification was 50×, 400×, 1,000×, 2,000×, 5,000×, 10,000×, and 100,000×, respectively. The corresponding scale bars were 445, 56, 22, 11, 4.4, 2.2, and 0.22 μm, respectively. **(D)** Comparison of soybean leaf hair. The surface structure of leaf hair was observed under SEM using soybean leaf tissue and the PSIT. From left to right, the magnification was 220×, 300×, 1,000×, 2,000×, 5,000×, 10,000×, and 100,000×, respectively. The corresponding scale bars are 100, 73, 22, 11, 4.4, 2.2, and 0.22 μm respectively.

To test whether the method can be applied to various plants and cell surfaces, we analyzed the surface structures of Arabidopsis, tobacco, rice, dragonfly wing, and soybean leaf trichomes (**Figure 4**). The sample preparation for PSIT was less complicated and time consuming than that for SEM. Furthermore, there were no obvious differences in image resolution obtained by PSIT or SEM.

In Arabidopsis, tobacco, and rice, we also studied stomatal aperture status using the PSIT (**Figure 4A**). The results showed that the imaging resolution of stomata using the PSIT was about 30–50 nm, which is sufficient to study stomatal aperture. This also indicated that PSIT could be effectively applied to the study of stomatal aperture in other higher plants. Further, we analyzed the surface papilla of the rice leaf using the PSIT (**Figure 4B**), in which the waxiness of the surface layer could be clearly observed. This result demonstrated that the highest resolution rate of the PSIT could reach approximately 50 nm. Moreover, we observed the lotus surface using the PSIT and observed significant protrusions, leaf nano-hairs (Balani et al., 2009), and waxiness (**Figure S1**).

Finally, to verify that there were no significant differences in the analytical precision between PSIT and traditional SEM, we compared the imaging of soybean leaf trichomes and dragonfly wings using these two methods (**Figures 4C, D**), which demonstrated no distinct differences in the resolution rate. However, there is more surface texture visible in the SEM images, whereas the PSIT images appear to show smoother surfaces (**Figure 4D**), which might be attributed to not completely dehydrating the soybean leaf trichomes by traditional SEM. Taken together, these results indicated that the precision of imaging by PSIT can satisfy demand for the study of plant stomata and cell surfaces.

## Discussion

By applying the most recent advancements in polyaddition silicone rubber nanomaterials, our approach can be used as a practical application to study and analyze plant stomata and the structure of plant cell surfaces. This novel PSIT can be easily applied to different plant cell surfaces and has high-resolution imaging performance. Furthermore, the results of stomatal aperture as measured by the PSIT could ideally correspond to the photosynthetic rate (**Figures 2B**, **3B** and **Figure S2**). In addition, compared with the g_s_ and stomatal aperture measured before and after making an impression (**Figure S3**), it demonstrated that our method is nondestructive for most plants, allowing the continuous dynamic variation of plant stomatal aperture to be studied in situ. In the future, PSIT may be applied to observe the surface structure of fossil flowers, fruits, and seeds (Friis et al., 2014; Friis et al., 2015) and create high-efficient photovoltaic devices (Huang et al., 2015). In short, with standard SEM, this PSIT can be widely and advantageously applied to many areas of plant, animal, and material sciences under nondestructive conditions.

## Data Availability Statement

All datasets generated for this study are included in the article/**Supplementary Material**.

## Author Contributions

YH, CL, JF, ZW, and DJ conceived and designed the experiments. YH, KZ, JF, ZW, and BL performed the experiments. YH and DJ analyzed the data. YH and DJ co-wrote the manuscript. YH, CL, and DJ supervised the project. All authors discussed the results and commented on the manuscript.

## Conflict of Interest

The patent applicant was supported by Zhejiang University. The patent title is “A method for obtaining the surface structure model of plant leaves”. The inventors include: YH, DJ, CL, ZW, BL, and Judy Jernstedt. The application number is ZL201510071346.5 (China) and the priority date is 11-Feb-2015. The method described in this manuscript was covered in the patent. The holder of this patent is Zhejiang University. The financial interests from the patent therefore belong to Zhejiang University.
