# Supplementary material for: Highly Efficient Nanoscale Analysis of Plant Stomata and Cell Surface Using Polyaddition Silicone Rubber
Source: Front Plant Sci. 2019 Dec 13;10:1569. doi: 10.3389/fpls.2019.01569 (PMC6923247; doi:10.3389/fpls.2019.01569)
Supplement: Supplementary file 1 [file DataSheet_1.pdf]

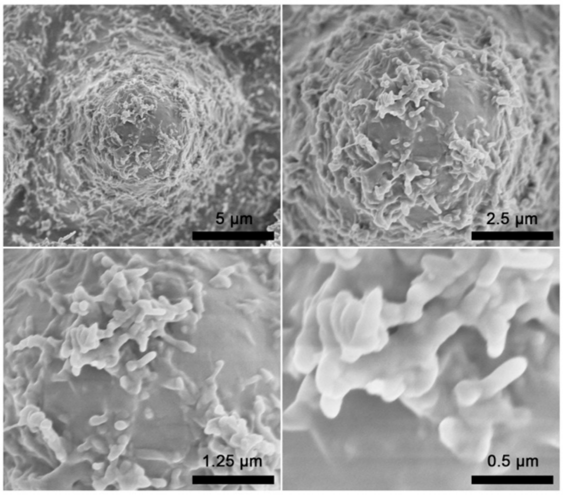

Figure S1. **The application of plant surface impression technique of (PSIT) in Lotus leaf.** The surface of lotus leaf was made by PSIT. The sections were observed under SEM by 5000×, 10000×, 20000× and 50000× magnifications respectively. Scale bars were 5 µm, 2.5 µm, 1.25 µm and 0.5 µm, respectively.

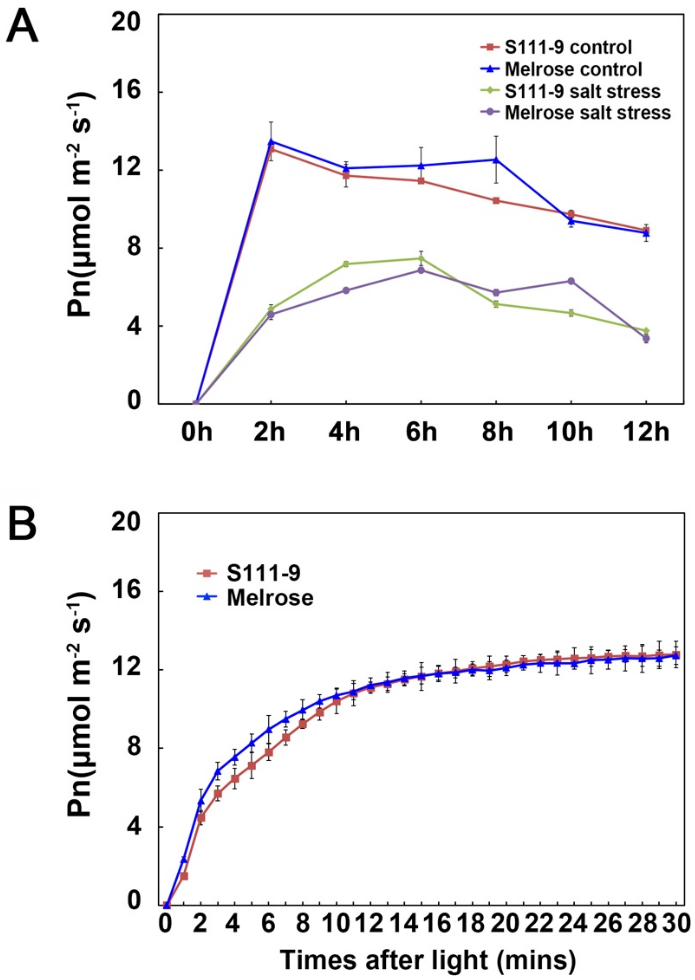

Figure S2. **The changes in photosynthetic rate under different salt stress and after light-illuminated.** (**A**) Effect of salt stress on photosynthetic rate (Pn). The third fully expanded leaves of 25-day old Melrose and S111-9 under no salt stress and 150 mM salt stress for 0 h, 2 h, 4 h, 6 h, 8 h, 10 h and 12 h were used in this experiment. (**B**) Changes in photosynthetic rate (Pn) after light-illuminated. The third fully expanded leaves of 25-day old Melrose and S111-9 under no salt stress for 0 mins, 5 mins, 10 mins, 15 mins, 20 mins and 25 mins were used in this experiment. Three biological replicates were done in this experiment.

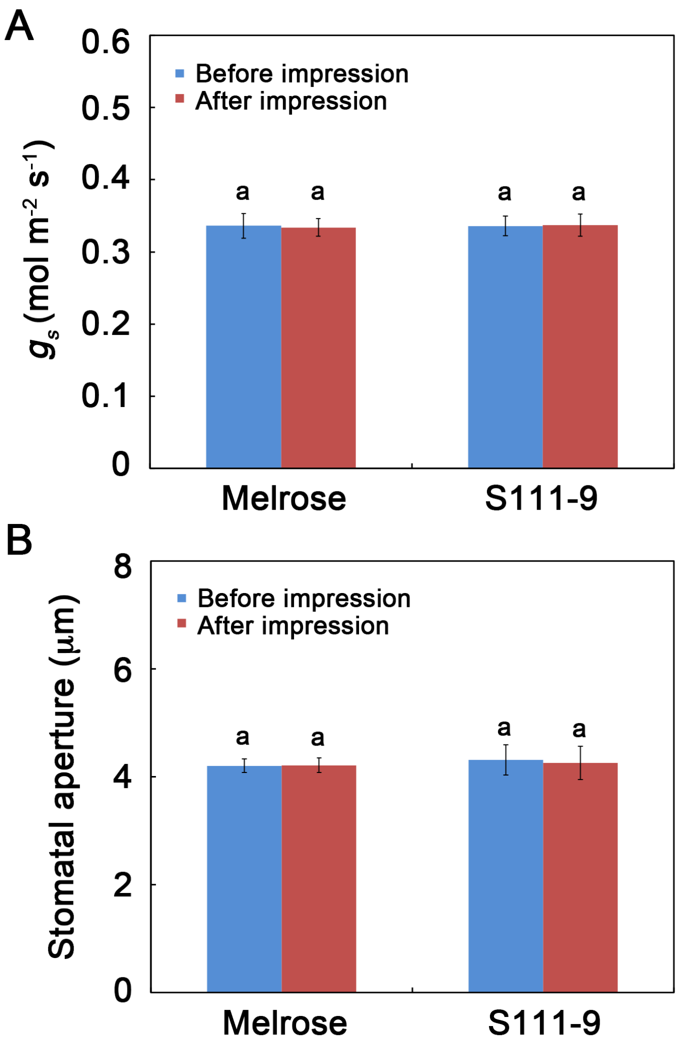

Figure S3. **Effects of PSIT on leaf's stomatal conductance and stomatal aperture.** (**A**) Effects of PSIT on leaf's stomatal conductance (g_s). The g_s were measured before and after making an impression by PSIT with a portable photosynthesis system (*Licor*-6400; LICOR Inc., Lincoln, NE, USA). The measurement method for g_s was the same as described in Figure 2. (**B**) Effects of PSIT on leaf's stomatal aperture. The determination of stomatal aperture was described in Figure 2. All measurements were repeated at three biological replicates for statistical analysis. Bars represent the mean ± SD of at three biological replicates. Different letters indicate a significant difference among varieties or treatment ($P < 0.05$) by Tukey's test.
